# The PRE‐registration podiatry: Accessible recruitment and employment (PREPARE) project: A commentary on the novel approach to the podiatry workforce crisis in Glasgow

**DOI:** 10.1002/jfa2.70014

**Published:** 2024-11-16

**Authors:** Pamela Price, Paul Higgins, David Lambert, Diane M. Dickson, Ruth Barn

**Affiliations:** ^1^ Department of Podiatry NHS Greater Glasgow and Clyde Podiatry Service Queen Elizabeth University Hospital Glasgow UK; ^2^ Department of Podiatry and Radiography School of Health and Life Sciences Glasgow Caledonian University Glasgow UK

**Keywords:** apprenticeship, education, workforce

## Abstract

The profession of podiatry is facing significant workforce challenges and urgent solutions are required to increase workforce capacity. Apprenticeship models are available in England but as yet, not available in Scotland. This paper explores the local context of the current workforce challenges and summarises a novel solution collaboratively developed in Glasgow. A collaborative initiative between NHS Greater Glasgow and Clyde and Glasgow Caledonian University developed the PRE‐registration Podiatry: Accessible Recruitment and Employment (PREPARE) Project. Creative use of vacancy savings are being used to employ a limited number of individuals on a whole‐time Band 5 Annex 21 basis at the start of their podiatry degree for the duration of their studies. Academic timetables were consolidated with no change to module or learning outcomes with candidates spending 3 days per week on academic studies and 2 days per week employed within the host podiatry service during term time. If academic milestones are achieved, substantive Band five opportunities are available once registration is achieved. This project opens opportunities for graduate level study to the support workforce and others not in a position to pursue traditional models of full‐time education. To date, two additional health boards in Scotland have adopted this model and it has the potential to be a national approach for Scotland. High applicant numbers were achieved demonstrating increased interest in podiatry from this approach. This project has the potential to have a transformative impact on educating and training the future workforce, potentially being adopted by other professions facing similar workforce challenges.

## 1 BACKGROUND

Numbers of people entering the podiatry profession are declining across the UK [1, 2]. The need to find innovative ways of increasing workforce capacity has been highlighted by several key documents including the Saks Report [3] and the Scottish Government's Allied Health Professions (AHP) Education and Workforce Review [4]. Between 2016 and 2019 podiatry joiner rates averaged 7.6% whilst leaver rates averaged 8.1% [2]. Moreover, podiatrists (alongside paramedics, occupational therapists, diagnostic radiographers and speech and language therapists) were highlighted in NHS England's Long Term Workforce Plan as one of the professions with the largest projected shortfall due to the education and training pipeline not keeping up with anticipated demand [2]. Unlike other AHPs, even with targeted interventions, medium term shortfalls are predicted for podiatry [2]. Current routes into the podiatry profession across the UK include undergraduate Honours degrees, pre‐registration Masters degrees or apprenticeship routes which are governed by overarching standards for apprenticeships [5]. However, not all options are available in all devolved nations and currently, AHP programmes do not have apprenticeship routes available in Scotland. In Scotland, undergraduate degrees are typically 4 years in length in contrast to 3 years elsewhere in the UK. The aim of this paper is to explore a locally developed solution to the current workforce crisis facing podiatry.

## 2 LOCAL CONTEXT

National recruitment and retention issues are further compounded for NHS podiatry in Glasgow by three additional factors, (i) an ageing workforce profile in NHS Greater Glasgow and Clyde (NHSGGC) Podiatry (one‐third of 164 podiatrists 50 years or over as of August 2023) [6]; (ii) increased demand for podiatry services due to an ageing population with multiple comorbidities [2, 7] and (iii) high levels of vacancy (12% in NHSGGC Podiatry in November 2023). Furthermore, in Scotland there are currently two institutions offering podiatry courses, Glasgow Caledonian University (GCU) and Queen Margaret University, therefore, training necessitates relocation to Glasgow or Edinburgh which in the current economic climate precludes some people from pursuing this training pathway. One advantage of studying in Scotland at the present time is the comparatively lower cost where tuition fees for the first higher education course are paid by the Scottish government (if residency criteria are met) [8] unlike other UK nations where tuition fees can be prohibitive.

## 3 THE PREPARE MODEL

**Aim:** To provide a sustainable solution to the workforce crisis in podiatry in Scotland and increase numbers into the profession.

**Method:** PREPARE trainees are employed on a whole‐time Band 5 Annex 21 basis from initiation of the podiatry degree at GCU until graduation; over the course of the 4 year degree, the payment of the trainees increases incrementally from 60% to 75% of the Band 5 salary [9] if they successfully achieve academic milestones. Following graduation, candidates will be offered a substantive Band 5 role once HCPC registered. With the support of senior management, creative use of vacancy savings was used to establish the PREPARE approach in 2023. This was made possible by collaboration with GCU whereby the academic timetable was condensed to three full days, rather than including half days over a 5 day week, leaving 2 days per week for service‐led duties during term time. No change to learning outcomes or degree content were made to facilitate this approach. During non‐term time, trainees are supported with study leave during assessment periods and service‐led duties 5 days per week for the remaining weeks.

Advantages: Unlike the preferred podiatry apprenticeship partner for NHS England, PREPARE aligns trainees with the traditional model of delivery linked to the GCU program. This increases the opportunity for students to integrate, learn and access peer support across the academic and clinical environments. Trainees are further enabled due to the long‐standing collaboration between GCU and NHSGGC as GCU's main placement provider and a well‐established clinical education team. Exposure to service during term time carries the expectation of trainees working toward learning outcomes of their relevant podiatry specific and clinical modules. During non‐term time, trainee exposure optimises application of the achieved learning outcomes across our service model. As trainees progress, their opportunities to apply commensurate knowledge and skill are nurtured across our teams to ensure an appropriate width and depth of exposure is gained across patient type and clinical need. Being an NHS employee provides the trainees with full employee benefits and the opportunity to earn and learn concurrently.

**Mitigation of risk:** Organisational risk to NHS services around non‐engagement and non‐progression is mitigated through the use of the annex 21 contract with a trainee's failure to meet academic milestones for progression likely leading to contractual termination. Risk to GCU was mitigated using two mechanisms, (i) a collaborative process in the PREPARE shortlisting to ensure admissions criteria were met prior to interview and (ii) in order to preserve the numbers of applicants via the traditional undergraduate route, those already in receipt of an offer were not eligible for PREPARE.

**Progress:** Four trainees were recruited to NHSGGC and employed from Aug 2023 and a further four recruited in 2024 and a commitment to employ four each year on an ongoing basis. The 2024 cohort were recruited at an earlier time point to facilitate greater immersion in local teams prior to starting the degree. Two further NHS Scotland health boards (NHS Fife and NHS Forth Valley) have adopted the PREPARE approach for the September 2024 intake. Ongoing evaluation of the project is planned to ensure an iterative approach is taken to enrich the opportunities offered to future cohorts and understand the impact of PREPARE.

## 4 HYBRID DELIVERY

GCU are committed to increasing accessibility of the podiatry degree, as well as supporting excellence in podiatry education, to meet workforce demands and have recently obtained approval to pursue a hybrid delivery model for remote and rural areas. During the Covid‐19 pandemic, many university courses moved to fully online at short notice. Healthcare students' experience of this approach was varied with the majority preferring to have a mixture of in‐person and online [10]. Whilst the pandemic provides an opportunity to evaluate the experience of online learning, it is difficult to separate the student experience from the lived experience of the pandemic with all that entails in terms of isolation from friends and family coupled with the impact on clinical environments during this time. Our model of hybrid delivery requires attendance on campus at scheduled weeks for in‐person practical training and synchronous attendance online for theoretical components for remote and rural students. A systematic review on synchronous hybrid learning summarised the key benefits of a more flexible, engaging learning environment compared to fully online or fully on‐site albeit with some limitations including additional work required by the facilitator to fully engage both remote and in‐person audiences simultaneously [11]. Offering a hybrid route removes geographical boundaries and financial constraints of relocation for people living in remote and rural areas. Even for candidates not employed via PREPARE, hybrid delivery is available to those residing rurally opening opportunities for supply of a local workforce to those areas unable to fill vacancies.

## 5 WIDER IMPLICATIONS AND CONCLUSIONS

The PREPARE approach affords several advantages for all stakeholders. For the PREPARE candidates, it (i) increases opportunities for adult learners and those who are not able to leave full‐time employment to access further study; (ii) opens up new opportunities for career progression and development for the support workforce to upskill and retrain; (iii) provides job security, employee benefits and guaranteed employment opportunities following graduation and (iv) provides candidates with opportunities for year‐round consolidation of academic learning in a workplace environment. Moreover, these opportunities for consolidation of academic theory and workplace learning enable the profession to respond to the rapidly changing healthcare environment [12].

For the profession, (i) the PREPARE trainees will have extensive clinical experience in excess of traditional numbers of clinical hours. During term time in year 1, PREPARE trainees gained an additional 60 days of clinical exposure linked to service delivery which enables development not only of podiatry specific knowledge and skill but also of trainee development associated with integration within clinical teams, being part of service delivery, understanding and bestowing the values of organisational citizens. (ii) Trainees will have a detailed knowledge of policies and procedures within the NHS and increased readiness to enter the workplace; (iii) during training trainees will help maintain safe workforce levels and safeguard frontline clinical services; (iv) widening access increases numbers of people entering the profession; and (v) this project has the potential to reduce the early career graduate attrition rates that are currently higher in podiatry than other AHPs [13]. Whilst numbers of applicants to podiatry are routinely lower than other AHPs such as physiotherapy, reasons for this disparity are unclear. Postulated reasons include lack of awareness of the profession and misunderstanding of the scope of practice. High applicant numbers were received for the PREPARE project in NHS GGC. In comparison to the numbers applying via the traditional route directly to GCU, PREPARE received in the region of 50% of that number highlighting an increased interest in the profession generated by this project. This is potentially suggestive that podiatry is an attractive career option when financial barriers to pursuing full time education are removed. Podiatry typically attracts a range of applicants from diverse backgrounds; less than 50% of the GCU student cohort are school leavers and a substantial proportion choose podiatry as a second career thus increasing accessibility provides greater opportunities to further widen access to a varied demographic.

The NHS England long term workforce plan set out ambitious targets for at least 80% of podiatrists to join the workforce via an apprenticeship route by 2031/32 [2]. With current political uncertainty in light of UK general elections and financial support for workforce solutions not readily available, it has been necessary to proactively create opportunities to tackle the prevailing issues. Furthermore, with the current economic pressures facing higher education institutions [14, 15], strategies are required to maintain the future of the profession as departments risk closure due to non‐viable programmes. The utility of PREPARE is not confined to podiatry; it is hoped that sharing this approach with other professions facing similar recruitment challenges will provide the catalyst to proactively influence their future workforce capacity. NHSGGC and GCU have worked collaboratively to have a unified approach for Scotland for podiatry and are keen to share learning with colleagues across the professions and national boundaries.

The PREPARE project has the potential to redefine how we recruit and train undergraduate podiatry students whilst facilitating retention of the workforce. PREPARE has demonstrated the potential to increase applicants to podiatry, affords opportunities for consolidation of learning with anticipated contribution to successful completion of pre‐registration training. The early, targeted investment in the development of trainees has the potential to enable them to become the graduate, specialist and advanced podiatrist of the future and meet the demands of 21^st^ Century podiatry.

## AUTHOR CONTRIBUTIONS

All authors prepared, reviewed, edited and approved the manuscript.

## CONFLICT OF INTEREST STATEMENT

The authors declare no conflicts of interest.

## PATIENT CONSENT STATEMENT

Not applicable.

## PERMISSION TO REPRODUCE MATERIAL FROM OTHER SOURCES

Not applicable.

## CLINICAL TRIAL REGISTRATION

Not applicable.

## ETHICS STATEMENT

Not applicable.

## Data Availability

Not applicable. Data sharing is not applicable to this article as no datasets were generated or analysed during the current study.
